# Epidemiology of carbapenem-resistant *Klebsiella pneumoniae* ST15 of producing KPC-2, SHV-106 and CTX-M-15 in Anhui, China

**DOI:** 10.1186/s12866-022-02672-1

**Published:** 2022-11-01

**Authors:** Hang Zhao, Zhien He, Yujie Li, Baolin Sun

**Affiliations:** 1 College of Life Science and Technology, Mudanjiang Normal University, Mudanjiang, China; 2 Department of Oncology, The First Affiliated Hospital, University of Science and Technology of China, Hefei, Anhui China; 3 School of Life Science and Medicine, University of Science and Technology of China, Hefei, Anhui China

**Keywords:** Carbapenem-resistant *Klebsiella pneumoniae*, ST15 *Klebsiella pneumoniae*, Antibiotic resistance, β-lactams, Whole genome sequencing (WGS), Virulence factors

## Abstract

**Background:**

It is well known that carbapenem-resistant *Klebsiella pneumoniae* (CRKP) has become a more problematic public health issue due to its widespread spread worldwide. In China, ST11-type CRKP is the most prevalent CRKP, but ST15-type CRKP, a recently prevalent high-risk clone, has emerged widely throughout China, posing a serious public health risk. Therefore, we conducted an epidemiological of an outbreak of ST15 CRKP of producing CTX-M-15, KPC-2 and SHV-106 in a tertiary hospital in Anhui, China, to Understanding the potential risks of the current STT15 CRKP outbreak.

**Results:**

From July 2021 to December 2021, 13 ST15 CRKP isolates were identified by collecting non-repeated clinical multidrug-resistant isolates, with all capsular typing of serotype KL19. All ST15 CRKP isolates were resistant to cephalosporins, carbapenems and quinolones, but were sensitive to amikacin, tigecycline and polymyxin B. In addition, isolates carried *bla*_SHV−106_ (100%), *bla*_KPC−2_ (69%), *bla*_CTX−M−15_ (69%), *bla*_TEM−1B_ (69%), *bla*_OXA−1_ (62%) and *bla*_LAP−2_ (8%), as well as iron chelators (*iutA*, *ybt*, *fyuA*, *ent*, *fepA*, *irp1*, *irp2*, 100%) were detected. In phenotyping experiments, all ST15 CRKP exhibited lower growth rates than NTUH-K2044, and all ST15 CRKP did not exhibit mucoviscositty characteristics. However, in the *Galleria mellonella* infection model, isolates 21081212, 21081241 and 21091216 were more lethal than the hypervirulent isolates NTUH-K2044. Sequencing results showed that the genetic environment surrounding the genes *bla*_SHV−106_, *bla*_KPC−2_, *bla*_CTX−M−15_, *bla*_OXA−1_ and *bla*_TEM−1B_ were all identical in the ST15 CRKP isolates. Phylogenetic analysis showed that 13 ST15 CRKP isolates were divided into three subgroups, and when placed in global analysis, 10 of them were highly homologous to isolates from Jiangsu, two were highly homologous to isolates from Zhejiang, and one was homologous to an isolate from an unlabelled region.

**Conclusion:**

Our research shows that ST15 CRKP, which carries multiple β-lactamases genes and siderophores-encoding genes, may be evolving to hypervirulence and may have spread widely in localised areas. Therefore, environmental surveillance and clinical infection control in hospitals should be strengthened to prevent further spread of ST15 CRKP.

**Supplementary Information:**

The online version contains supplementary material available at 10.1186/s12866-022-02672-1.

## Background

One of the most prevalent nosocomial infections globally, *Klebsiella pneumoniae* is an Enterobacteriaceae bacterium that frequently infects elderly patients, patients in intensive care units and immunocompromised patients in hospitals [1]. In recent years, the prevalence of resistant *K. pneumoniae* has become more widespread due to the overuse of antibiotics, leading to the emergence of multidrug-resistant (MDR) isolates through the acquisition of different resistance genes during the transmission process [2]. Subsequently, carbapenems (e.g. imipenem, meropenem and doripenem) have become the last line of defense in the treatment of *K. pneumoniae* infections [3]. Unfortunately, due to the spread of mobile genetic elements has led to a steady increasing in CRKP [4]. The World Health Organisation (WHO) issued a report in 2017 highlighting the serious threat posed by carbapenem-resistant *K. pneumoniae* (CRKP) [5]. According to the data from China antibiotic resistance surveillance system (http://www.carss.cn/), the average detection rate of CRKP in China was 10.9% in 2020, and the detection rate reached more than 30% in some areas, and the trend is slowly increasing. In addition, CRKP infections have led to increased treatment difficulties with mortality rates as high as 40–50%, which poses a significant public health challenge [6].KPC-type β-lactamase is one of the important carbapenemases of CRKP and is widely seen in clinical conditions [7]. In China, a study showed that the sequence type of CRKP is predominantly the KPC-producing ST11 type [8]. ST15 *K. pneumoniae* is an emerging high-risk clonal isolate with frequent hospital outbreaks and has emerged carrying virulence-resistant heterozygous plasmids and ESBL genes [9]. CTX-M-15 β-lactamase, on the other hand, poses a significant threat to most antibiotics, especially cephalosporins [10]. SHV-type β-lactamases are thought to be present in isolates with little effect [11]. SHV-106-type β-lactamase was first reported in Portugal in 2009 [12]. Location-specific outbreaks of carbapenemase and β-lactamases carrying associated ST15 *K. pneumoniae* may be highly homologous. Still, the variability in antibiotic resistance of ST15 *K. pneumoniae* isolates from different study sites suggests that the potential for ST15 *K. pneumoniae* to acquire different resistance genes is substantial [13].

Here, we aimed to phenotypically and genomically characterise ST15 CRKP collected from a tertiary hospital in Anhui, China, to further explore the potential risk of ST15 CRKP outbreaks, including antibiotic resistance, virulence, etc.

## Results

### Clinical characteristics

According to multilocus-sequence typing (MLST) and capsular typing, 13 clinical isolates of *K. pneumoniae* were ST15-type and all were serotype KL19. Thirteen isolates of ST15 CRKP were from sputum (n = 7), blood (n = 4), urine (n = 2), of which 84.6% (n = 11) were male and 15.4% (n = 2) were female, age ≥ 60 y was 61.5% (n = 8) and, significantly, age ≤ 45 y was 15.4% (n = 2). In addition, the majority of isolates were from the ICU (n = 10).

### Phenotyping experiments

**Fig. 1:**
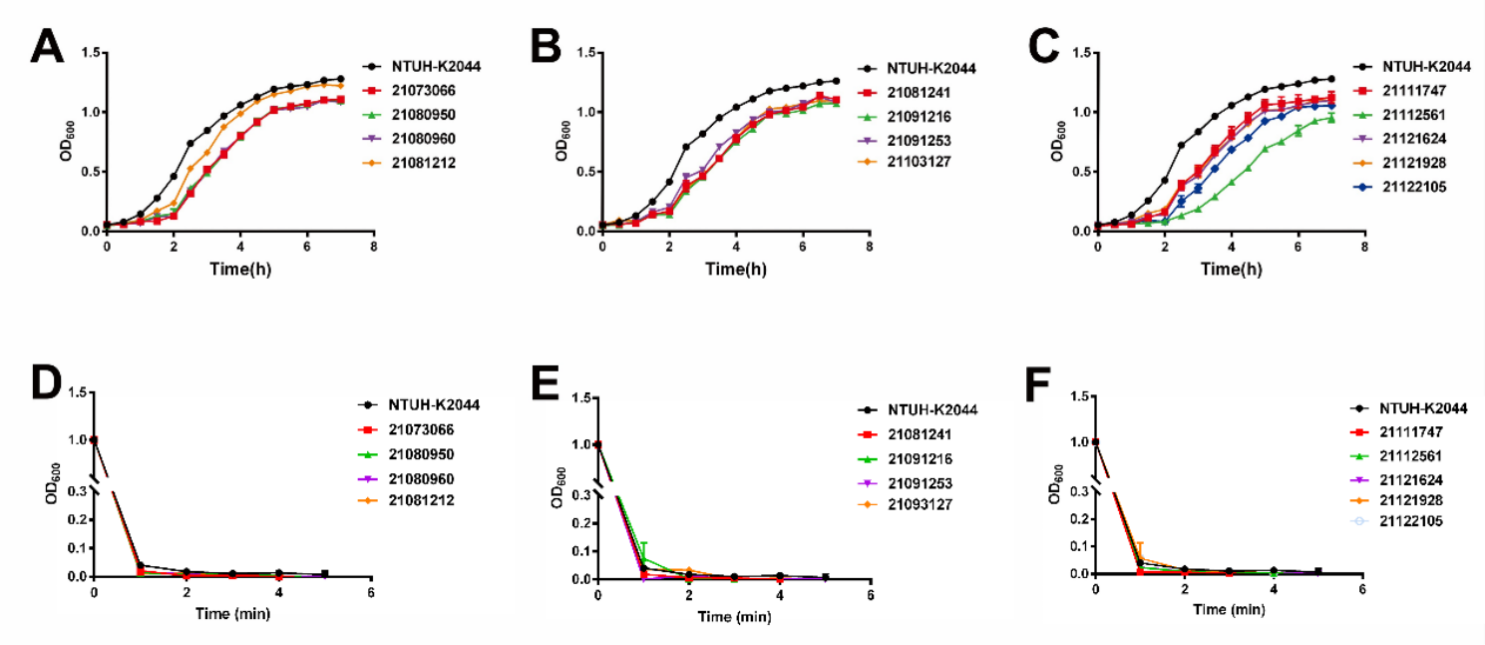
Growth curves and mucoviscosity assay of ST15 CRKP isolates. Establishment of growth curves in LB medium to assess the adaptability of ST15 CRKP isolates to the environment (A), (B), and (C) and determination of capsular polysaccharide viscosity using mucoviscosity assay (D), (E), and (F). Hypervirulent isolates NTUH-K2044 was used as a control. CRKP, carbapenem-resistant *Klebsiella pneumoniae*

**Fig. 2:**
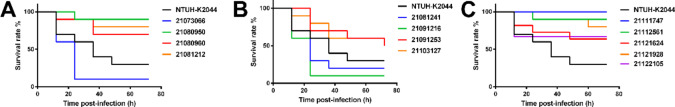
ST15 CRKP isolates exhibit a high fatality rate in the *G. mellonella* infection model. *G. mellonella* infection model for evaluating the virulence of ST15 CRKP isolates. Nonhypervirulent ST15 CRKP isolates 21073066, 21081241 and 21091216 exhibit higher mortality than the hypervirulent isolates NTUH-K2044 in *G. mellonella* infection model (A), (B), and (C). CRKP, carbapenem-resistant *Klebsiella pneumoniae*

When comparing growth curves, mucoviscosity assay results, and a *G. mellonella* infection model, we used the hypervirulent isolate NTUH-K2044. In terms of growth rate, NTUH-K2044 showed the fastest growth rate (Fig. 1 A–C). The other ST15 CRKP isolates displayed distinctly weaker growth rates than NTUH-K2044, while 21081212 displayed a growth rate that was comparable to that of NTUH-K2044. Overnight cultures were centrifuged at low speed for 5 min, and all isolates, including NTUH-K2044, were completely clear in the supernatant at 3 min (Fig. 1D–F). In *G. mellonella* infection model, isolates 21081212, 21081241 and 21091216 had higher lethality than NTUH-K2044 (Fig. 2 A–C).

### Antibiotic resistance profiling

All ST15 CRKP were resistant to amoxicillin/clavulanic acid, ceftriaxone, ceftazidime, cefoperazone/sulbactam, cefuroxime axetil, cefuroxime sodium, cefepime cefoxitin, ertapenem, imipenem, levofloxacin, and piperacillin/tazobactam (except 21122105 which was sensitive to imipenem, minimum inhibitory concentrations [MIC] = 0.25 mg/L), but was sensitive to amikacin, polymyxin B, and tigecycline. In addition, isolates 21081212, 21081241, 2109125 and 21121928 were resistant to trimethoprim/sulfamethoxazole. The distribution of antibiotic resistance is shown in **Additional file 1**.

### Antibiotic resistance genes (ARGs)

**Fig. 3:**
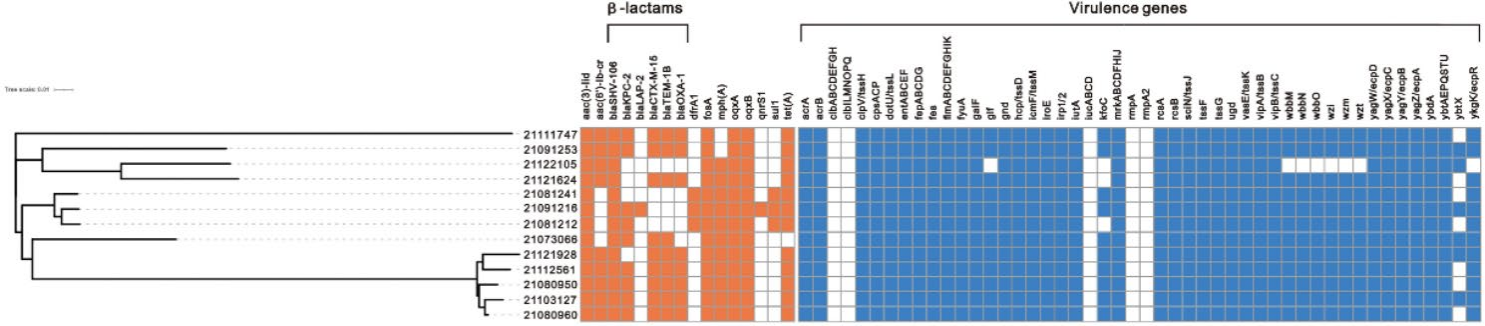
Distribution of resistance genes and virulence factors of ST15 CRKP isolates. The phylogenetic showed that the ST15 CRKP isolates were divided into three subgroups. The resistance gene colour is labelled orange and the virulence factor is blue. Blank spaces indicate that the gene is not present. CRKP, carbapenem-resistant *Klebsiella pneumoniae*

The distribution of resistance genes and virulence factors is shown in Fig. 3. A total of 16 resistance genes were detected in all ST15 CRKP, six of which were β-lactams. All ST15 CRKP carried the *bla*_SHV−106_ gene (100%). In addition, the isolates carried the carbapenemases-encoding genes *bla*_KPC−2_ (69%), the β-lactamases-encoding genes *bla*_LAP−2_ (8%), *bla*_TEM−1B_ (69%) and *bla*_OXA−1_ (62%), and the ESBL-encoding genes *bla*_CTX−M−15_ (69%). Furthermore, while *bla*_KPC−2_ was not present in all ST15 CRKP isolates, but all ST15 isolates were Ertapenem and Imipenem resistant (except 21122105). We also found that all ST15 CRKP isolates carried the gene *oqxAB* encoding the quinolone efflux pump and the phosphomycin resistance gene *fosA* [14]. According to previous studies, *oqxAB* encodes the efflux pump responsible for quinolone resistance, but it cannot be excluded that cephalosporins, carbapenems, and other antibiotics are pumped from the *K. pneumoniae* cell membrane through this efflux pump [15], while *fosA* may be widespread in gram-negative bacteria, which is consistent with previous studies [16]. Also tetracycline resistance gene *tet(A)* (92%), aminoglycoside resistance genes *aac(3)-lid* (100%) and *aac(6’)-lb-cr* (69%). In addition, the methicillin resistance gene *dfhrA1*, the sulfonamide resistance gene *sul1*, and the macrolide resistance gene *mph(A)* were also present in a few isolates.

### Virulence factors

We found siderophore coding genes in all ST15 CRKP, including the aerobactin-encoding genes (*iutA*, 100%), yersiniabactin-encoding genes (*ybt*, *irp1*, *irp2*, *and fyuA*, 100%), and enterobactin-encoding genes (*ent*, *fepA*, 100%). In addition, the multidrug efflux pump *acrAB* was present in all ST15 CRKP. The hypervirulence-defining genes *iucA*, *iroB*, *peg-344*, *rmpA*, and *rmpA2* were not found in all ST15 CRKP isolates, but the lethality of 21073066, 21081241 and 21091216 was distinctly higher than that of the hypervirulence NTUH-K2044 in the *G. mellonella* infection model. In previous studies, *G. mellonella* infection models were frequently used to determine hypervirulent isolates, but because of the phenomenon of apparent overlap in virulence, high *G. mellonella* lethality does not represent hypervirulence and is not accurate in determining whether it is hypervirulent *Klebsiella pneumoniae* (hvKP) [17]. In our study, no-hypervirulent isolates also exhibited higher *G. mellonella* lethality.

### Analysis of mobile genetic elements

**Fig. 4:**
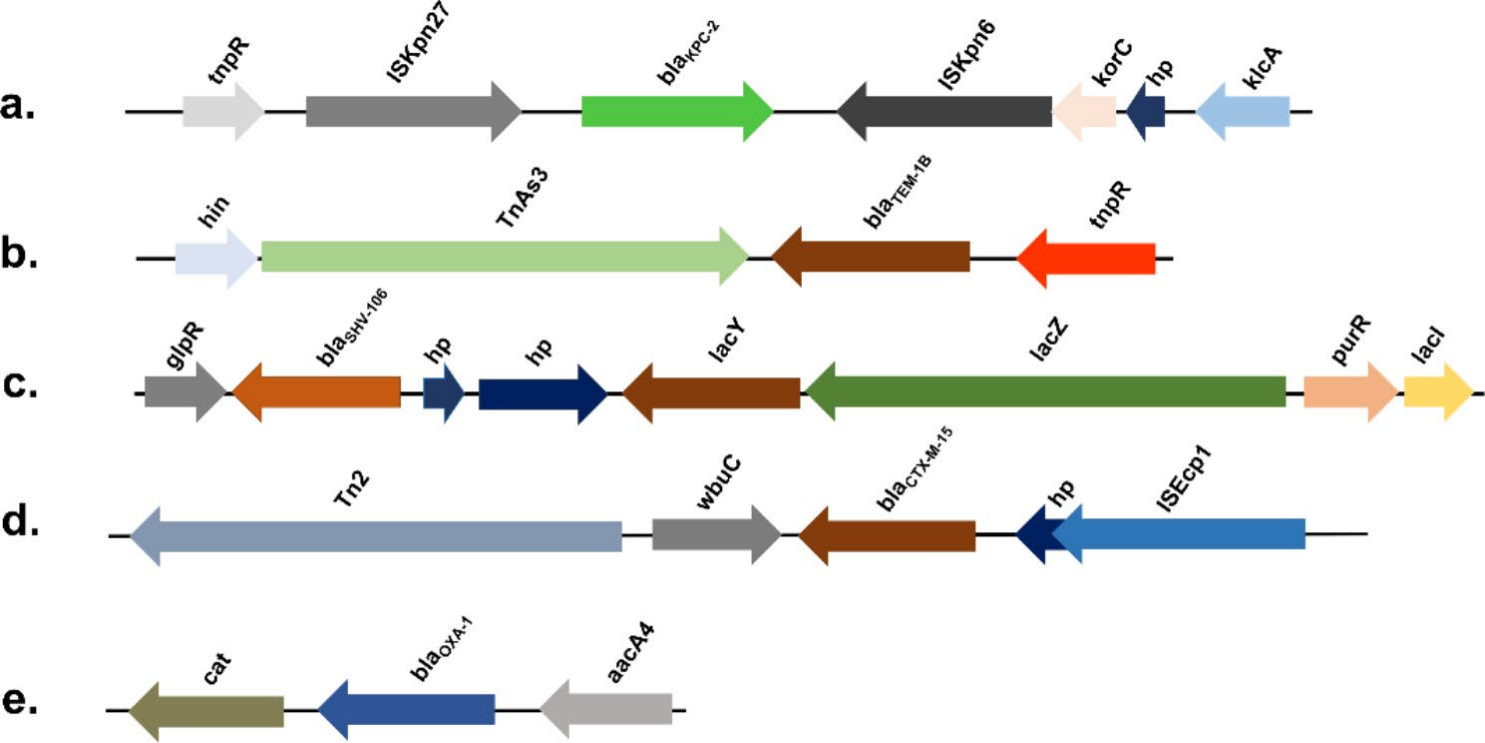
The mobile genetic elements of the *bla*_KPC−2_, *bla*_TEM−1B_, *bla*_SHV−106_, *bla*CTX-M-15, and *bla*_OXA−1_ gene of ST15 CRKP are identical. (a) *tnpR*-IS*Kpn27*-*bla*_KPC−2_-IS*Kpn6*-*korC*-hp-*klcA*. (b) *hin*-Tn*As3*-*bla*_TEM−1B_-*tnpR*. (c) glpR-*bla*_SHV−106_-hp-hp-*lacY*-*lacZ*-*purR*-*lacI*. (d) Tn2-*wbuC*-*bla*_CTX−M−15_-hp-IS*Ecp1*. (e) *cat*-*bla*_OXA−1_-*aacA4*. CRKP, carbapenem-resistant *Klebsiella pneumoniae*; hp, hypothetical protein

The sequencing results showed that the insertion elements and transposons of *bla*_SHV−106_, *bla*_KPC−2_, *bla*_CTX−M−15_, *bla*_OXA−1_, and *bla*_TEM−1B_ genes were identical for all isolates (Fig. 4). Both *bla*_SHV−106_ are located on chromosomes and inserted into the downstream of lactose operon lac, forming glpR-*bla*_SHV−106_-hp-hp-*lacY*-*lacZ*-*purR*-*lacI* structure. The upstream and downstream of *bla*_KPC−2_ are then composed of IS*Kpn27* and IS*Kpn6*. *bla*_CTX−M−15_ forms a Tn2-*wbuC*-*bla*_CTX−M−15_-hp-IS*Ecp1* with IS elements. *bla*_OXA−1_ forms the *aacA4*-*bla*_OXA−1_-*cat* structure. *bla*_TEM−1B_ forms the hin-Tn*As3*-*bla*_TEM−1B_-*tnpR* structure.

### Phylogenetic analysis

**Fig. 5:**
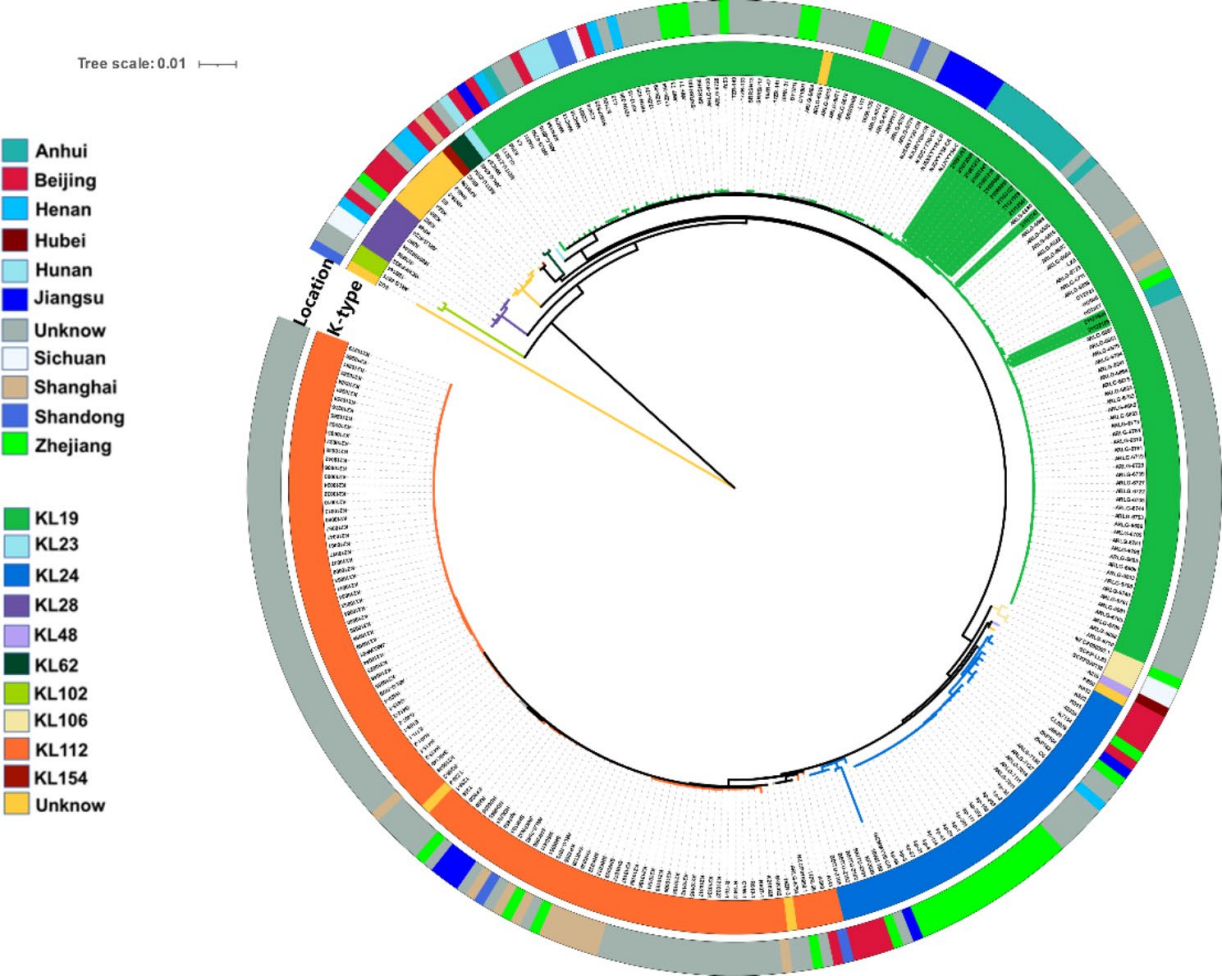
Phylogenetic construction of 287 isolates of ST15 *K. pneumoniae* based on core genomic SNPs using Harvest v1.1.2 and further visualisation and modification in iTOL v4. The colour of the outer ring indicates the different regions of the isolates, and the inner ring indicates the different capsular serotypes. The ST15 CRKP in this study is marked in green and other ST15 *K. pneumoniae* from the Chinese collection of the NCBI database. CRKP, carbapenem-resistant *Klebsiella pneumoniae*

By using BacWGSTdb 2.0 for comparative analysis of 13 ST15 CRKP isolates, single nucleotide polymorphism (SNP) analysis showed a maximum of 993 (21111747 and 21121928) and a minimum of 21 (21080950 and 21103127) (**Additional file 2**). For the 13 isolates, we constructed a phylogenetic using maximum likelihood based on the core genomic SNPs (Fig. 3), and the results were divided into three subgroups, 21121624, 21091253 and 21122105 with highly homology, 21121928, 21080950, 21080960, 21103127 and 21112561 with highly homology, and 21,081,212, 21,081241 and 21,091,216 with highly homology. To further investigate the genetic relationships of ST15 CRKP isolates, we constructed a global phylogenetic (Fig. 5) using 287 *K. pneumoniae* from the published genome-wide series of ST15 isolates from China (NCBI), and because there were fewer data available for ST15 isolates, we included all ST15 sequences after excluding unassembled genomes, although they carried different resistance genes. 21121624 and 21122105 are highly homologous to isolate HD006 from Zhejiang, which is located in the same evolutionary branch. 21111747 is highly homologous to isolate ARLG-6699 from an unlabelled region. The other 10 ST15 CRKP isolates in this study were closest to the isolate NJJYY54-C from Nanjing, Jiangsu, which produced KPC-2 and SHV-28.

## Discussion

KPC-2-producing *K. pneumoniae* was first reported in China in 2007 [18], and since then it has become widespread in China as well as in several countries and regions [19]. It is widely believed that KPC-2-producing ST11 CRKP is the most prevalent isolates in China. However, according to a study, the Chinese ST sequence type is currently shifting from ST11 to ST15, and ST15 showed higher resistance in serocidal assays [20]. ST15 *K. pneumoniae* is an emerging high-risk clone that has become a serious public health risk and has led to the emergence of ST15 hvKP with the spread of virulent and drug-resistant heterozygous plasmids [9]. In addition, with the widespread use of carbapenems, ST15 CRKP has emerged in recent years in China [21], India [22], Japan [23], and Iran [24] in several places. In this study, we collected 13 ST15 CRKP isolates from July 2021 to December 2021, with more male patients, and the most frequent source of isolates was sputum. Most of the patients had a history of prolonged hospitalisation, invasive procedures and antibiotic therapy, which significantly increased the risk of CRKP fixation [25]. The most frequent source of isolates was still the ICU ward, suggesting that immunocompromise is still the leading cause of infection in patients. It also suggests that hospitals should strengthen in-hospital ward supervision to prevent transmission between patients and health care workers.

In terms of antibiotic resistance, all ST15 CRKP have extremely high rates of resistance to common antibiotics, including amoxicillin/clavulanic acid, ceftriaxone, ceftazidime, cefoperazone/sulbactam, cefuroxime axetil, cefuroxime sodium, cefepime cefoxitin, ertapenem, imipenem, levofloxacin, and piperacillin/tazobactam, This is the same result found in a previous study of ST11 *K. pneumoniae*. Still, it is noteworthy that all ST15 in this study belonged to MDR, which is significantly higher than ST11 [26]. Meanwhile, according to antibiotic resistance results, we should use antibiotic treatment rationally. ST15 CRKP showed low resistance to amikacin, which is consistent with previous studies [27]. In addition, unsurprisingly, all ST15 CRKP were sensitive to tigecycline and polymyxin B. Regarding phenotypic experiments, we found that ST15 grew slower than NTUH-K2044, which may be related to the presence of siderophores or virulence factors [28]. Mucoviscosity is due to *rmpA*-regulated *rmpD* production, a phenotype associated with hvKP isolates [29]. The ST15 CRKP isolate did not detect *rmpA* and *rmpD*. However, it is interesting to note that ST15 appears exceptionally lethal in the *G. mellonella* infection model, which we considered to be due to the presence of overlapping virulence, but whether there are other reasons for this remains to be investigated.

When we analysed the whole genome, the ST15 CRKP isolate carried multiple β-lactamases, including *bla*_KPC−2_, *bla*_CTX−M−15_, *bla*_SHV−106_, *bla*_TEM−1B_, and *bla*_OXA−1_. Most isolates carried several β-lactamases genes simultaneously, which is consistent with previously reported results that *bla*_TEM_, *bla*_SHV_, and *bla*_CTX−M_ (any two or all three) were present simultaneously in *K. pneumoniae* isolates [15]. OXA-producing ST15 was also found in other regions of China [30]. According to a study, the most common sequences producing ESBL are ST11 and ST15. In our research, ST15 was associated with carbapenemases and ESBL, making treatment more complex [31]. To our knowledge, *bla*_SHV−106_ has been rarely reported elsewhere after being registered only in Portugal. However, all STT15 CRKP carried *bla*_SHV−106_ in our study. Although SHV-type β-lactamases have been claimed to have little effect on the isolates in some studies, we still need to pay attention to SHV-type β-lactamases [12]. KPC-2 is an important carbapenemase, and the main clone of carbapenem-resistant hypervirulent *K. pneumoniae* (CR-hvKP) has been reported to be a KPC-2-producing ST11-type sequence in China [32]. However, 69% of ST15 in our study carried KPC-2, and reports suggest that hypervirulent KPC-2-producing ST15 occurs in China [33]. Interestingly, not all isolates carry *bla*_KPC−2_, but are resistant to carbapenems, and it is possible that a key role is played by the efflux pump encoded by *oqxAB*, which is increasingly prevalent in Enterobacteriaceae. Additionally, *oqxAB* may contribute to the resistance of *K. pneumoniae* isolates to tigecycline [34]. We found that all ST15 CRKP carry *fosA*, and the concomitant relevance of *fosA* is higher in KPC-2-producing *K. pneumoniae*. The combination of *fosA* and *bla*_KPC−2_ carried by plasmids accelerates the spread of resistance genes, and therefore fosfomycin should be used with caution for treating CRKP [35]. ESBL is the main cause of resistance to β-lactam antibiotics, and CTX-M-type enzymes have replaced TEM and SHV as the most common ESBL [11]. CTX-M-15 differs from CTX-M-3 in that a single amino acid is mutated and exhibits increased resistance to ceftazidime [36]. According to studies, ST15 CRKP often carries *bla*_CTX−M−15_ [27], and in the present study, 69% of ST15 CRKP isolates carried *bla*_CTX−M−15_. In addition, OXA-1 was another common β-lactamase in this study. OXA-1 type enzymes play an important role in hydrolysis of cefepime [37].

For virulence factors, we found that ST15 CRKP carried genes encoding aerobactin, enterobactin, and yersiniabactin, including *iutA, ybt, irp1, irp2, fyuA, ent, fepA*, that are essential for bacterial growth. According to previous studies, siderophores systems, such as aerobactin, yersiniabactin, and enterobactin, are more common in invasive isolates than in non-invasive isolates [38]. Although yersiniabactin was detected in nearly 30% of the non-invasive isolates and almost 80% of the bacteraemia isolates, we detected it in 100% of the ST15 CRKP. On the other hand, the ICE*kp* locus, where *ybt* is located, is a common mobile genetic element of *K. pneumoniae* [39]. The efflux pump, *acrAB*, is also critical for acquiring antibiotic resistance in gram-negative bacteria. Tigecycline and ciprofloxacin could be effectively removed by the efflux pump *acrAB*. However, carbapenems, some cephalosporins, and aminoglycosides cannot be removed by the pump [40]. We further analysed the genetic environment of the resistance genes. We found that the genetic environment around *bla*_KPC−2_, *bla*_SHV−106_, *bla*_CTX−M−15_, *bla*_OXA−1_, and *bla*_TEM−1B_ are identical in ST15, upstream, and downstream of *bla*_KPC−2_ are IS*Kpn27* and IS*Kpn6*, suggesting that the resistance genes may be transmitted via plasmids [41]. We found that the ST15 CRKP isolates formed three subgroups when we performed a phylogenetic analysis, implying that there may be clonal transmission between them. ST15 CRKP isolates were shown to be highly homologous to isolates from Jiangsu and Zhejiang in further analysis, suggesting that ST15 CRKP may have spread widely in East China.

There were limitations to this research, firstly, we were unable to consistently collect more ST15 CRKP from this hospital and secondly, we did not analysis the types of plasmids carried by ST15 CRKP due to the limitations of using second generation sequencing technology.

## Conclusion

In conclusion, we report outbreak of ST15 CRKP in a tertiary hospital in Anhui, China, which was phenotypically and genomically analysed. ST15 CRKP isolates are extremely resistant to commonly used antibiotics and carried multiple β-lactamase genes, and carried multiple siderophores encoding genes and *acrAB* and *oqxAB* efflux pumps, which may play an essential role in the virulence and resistance of ST15 CRKP isolates. Additionally, it is suggested by the phylogeny that ST15 CRKP may have spread widely in localised areas. Therefore, nosocomial MDR and carbapenem-resistant *K. pneumoniae* should be continuously monitored to prevent their evolution to highly virulent resistance through the acquisition of virulence factors carried by plasmids or removable genetic elements.

## Methods

### Sources and identification of bacterial isolates

A total of 13 MDR *K. pneumoniae* isolates from different patients from the clinical microbiology laboratory of Anhui Provincial Hospital (Anhui, China) were collected between July 2021 and December 2021. The VITEK 2 Compact System (bioMérieux, France) was used to identify the isolates. All isolates were processed according to standard operating procedures, scribed in a sterile environment onto 5% sheep blood agar plates and cultured at 37 °C.

### Antibiotic susceptibility testing

The MICs of Amikacin, Amoxicillin/Clavulanic acid, Ceftriaxone, Ceftazidime, Cefoperazone/Sulbactam, Cefuroxime axetil, Cefuroxime sodium, Cefuroxime, Cefepime, Cefoxitin, Ertapenem, Imipenem, Levofloxacin, Piperacillin/Tazobactam, Trimethoprim/Sulfamethoxazole were determined by the VITEK 2 Compact System and the results of the antimicrobial susceptibility tests were interpreted according to the Clinical Laboratory Standards Institute (CLSI) [42]. As previously described [43], the MIC of polymyxin B and tigecycline was determined by broth dilution using Mueller-Hinton broth and the results were analysed according to The European Committee on Antimicrobial Susceptibility Testing (EUCAST, https://www.eucast.org/).

### Growth curves

To study the environmental adaptability of the ST15 CRKP isolates, growth curves were measured in LB medium. As previously described [44], overnight cultures were diluted to 0.02 OD_600_/mL and 150 µL was pipetted into 96-well plates and incubated at 37 °C, measured every 0.5 h using an enzyme marker and recorded until the curve plateaued.

### Mucoviscosity assay

To study the viscosity of ST15 CRKP capsular polysaccharide, we measured its supernatant by low speed centrifugation [45]. Overnight cultures were diluted to 1 OD_600_/ mL and centrifuged at 2, 350 x *g* for 5 min and the supernatant was measured at OD_600_ per minute.

### ***Galleria mellonella*** infection model

Assessment of virulence of ST15 CRKP isolates using *G. mellonella* infection model [46]. Select *G. mellonella* larvae that are between 250 and 300 mg in size and free of pathogens for reserve (Tianjin Huiyude Biotechnology Co., Ltd.). Overnight bacterial cultures were diluted to 10^8^ CFU/mL using sterile saline, and 10 µL of diluted bacterial solution was injected into the centre of the second gastropod of the larvae using a microsyringe (100 µL). Sterile saline and the hvKP isolate NTUH-K2044 were injected as negative and positive controls, respectively. After injection, larvae were incubated in the dark at 37 °C and mortality was observed and recorded at 12 h intervals for 72 h. Three parallel groups were set up for each isolates.

### Capsular serology typing and MLST

Capsular typing was performed by PCR amplification using *wzi* PCR primers, MLST was performed by PCR amplification of seven pairs of housekeeping genes (*rpoB, gapA, mdh, pgi, phoE, infB* and *tonB*) of *K. pneumoniae*, ST sequences and capsular typing are available for comparison in the database (http://bigsdb.web.pasteur.fr).

### Whole genome sequencing (WGS), assembly, and annotation

WGS of *K. pneumoniae* was performed using an Illumina HiSeq 4000 platform in Genewiz (Anhui, China). Firstly, whole genomic DNA was extracted from the samples to be tested and libraries were constructed for cluster preparation and sequencing. The raw sequencing data (Pass Filter Data) was removed using cutadapt version 1.9.1 [47] to remove junctions and low quality sequences, followed by k-mer analysis using Velvet version 1.2.10 [48], and de Brujin plots were constructed using the overlap between k-mer. The scaffold sequences were further assembled from the contig sequences using pairwise relationships between Paired end reads and insert size distances using SSPACE version 3.0 [49]. Finally, GapFiller version 10 [50] was used to match all the library sequencing reads back to the scaffold sequence, and the gaps in the scaffold sequence were completed using the matched reads, and the scaffold sequence was extended. Genome assembly by unicycler version 0.4.9 [51] and annotated using the rapid prokaryotic genome annotation tool, Prokka version 1.14.6 [52].

### Genome analysis and phylogenetic construction

Acquired ARGs were identified using ABRicate version1.0.1 (https://github.com/tseemann/abricate) by aligning the genomic sequences to the ResFinder database [53]. The virulence factors of the isolates were identified using ABRicate version 1.0.1 by aligning the genomic sequences to the VFDB database [54]. The typing results were further determined using multilocus-sequence typing was performed by MLST 2.1 (https://cge.cbs.dtu.dk/services/MLST/). The HarvestTools kit (Parsnp, Gingr and HarvestTools) [55] and BacWGSTdb [56] were used to perform a comparative genomic analysis and phylogenetic analysis on the different isolates, and further visualisation and modification in iTOL v4 (http://itol.embl.de/) [57].

### Statistical analysis

Growth curves, mucoviscosity assay, and *G. mellonella* infection model data were statistically analysed and expressed as mean standard deviation or percentage using GraphPad Prism 7.0 (https://www.graphpad.com/). All experiments were repeated three times independently.

## Electronic supplementary material

Below is the link to the electronic supplementary material.

Supplementary Material 1

Supplementary Material 2

## Data Availability

The datasets used during the current study are available from the corresponding author upon reasonable request. Whole Genome Sequencing(WGS) has been uploaded to NCBI with the BioProject accession number PRJNA823907.

## List of abbreviations

CRKP: Carbapenem-resistant *Klebsiella pneumoniae*
WGS: Whole genome sequencing
MDR: Multidrug resistant
ESBL: Extended-spectrum beta-lactamases
WHO: World Health Organisation
MLST: Multilocus-sequence typing
hvKP: Hypervirulent *Klebsiella pneumoniae*
ckp: classical *K. pneumoniae*
PCR: Polymerase chain reaction
