# Supplementary material for: Epidemiology of carbapenem-resistant Klebsiella pneumoniae ST15 of producing KPC-2, SHV-106 and CTX-M-15 in Anhui, China
Source: BMC Microbiol. 2022 Nov 1;22:262. doi: 10.1186/s12866-022-02672-1 (PMC9624029; doi:10.1186/s12866-022-02672-1)
Supplement: Supplementary file 2 — Supplementary Material 2 [file 12866_2022_2672_MOESM2_ESM.docx]

**Supplementary Figure**

**
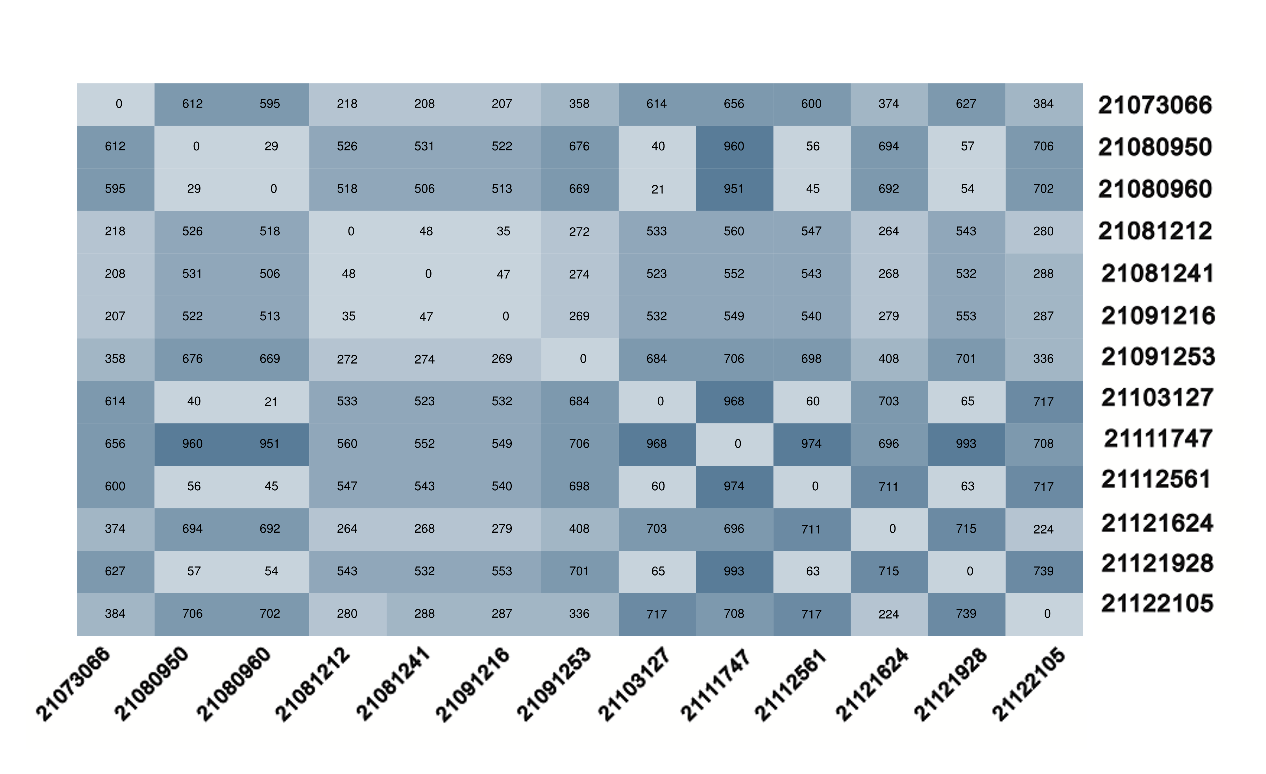
**

**Supplementary Figure 1.** SNP distribution of ST15 CRKP isolates.

Heatmap of the SNP distribution of ST15 CRKP isolates drawn using BacWGSTdb 2.0, with the color deepening with increasing SNPs.
